# Optimized maximum voluntary exertion protocol for normalizing shoulder muscle activity

**DOI:** 10.1080/23335432.2017.1308835

**Published:** 2017-04-07

**Authors:** Alison C. McDonald, Michael W. L. Sonne, Peter J. Keir

**Affiliations:** a Occupational Biomechanics Laboratory, Department of Kinesiology, Ivor Wynne Centre, McMaster University, Hamilton, Canada

**Keywords:** Shoulder muscles, maximum voluntary exertion, EMG, normalizing

## Abstract

Muscle activity is typically normalized to maximal activation from isometric maximum voluntary exertions (MVE) in posture and direction specific exertions for each muscle. This is challenging for the shoulder complex due to the large number of muscles. The objective of this investigation was to compare maximum shoulder muscle activity elicited from a multi-muscle MVE test protocol versus individual muscle MVE tests and determine their reliability. Ten healthy males had muscle activity recorded from 12 trunk and upper extremity muscles while performing 3 repetitions of 12 individual and 4 multi-muscle MVEs. Peak surface EMG (sEMG) amplitudes were compared using paired sample t-tests between the two protocols for each muscle. Individual muscle test maximum sEMG amplitudes differed significantly from the multi-muscle test protocol in 3 of 12 muscles (*p* < 0.05). In muscles that did not attain statistical significance, maximum amplitude differences of 6–15% were found. There was high reliability (Interclass Correlation Coefficient, ICC = 0.831–0.986) and no significant differences between the second and third repetitions of the protocol. Since differences of 6–15% could have functional significance, 8 MVE tests (3 multi-muscle, 5 individual muscle) were selected for future use. Using two repetitions of the reduced MVE protocol will reduce time, risk of pain and injury during experiments.

## Introduction

Surface electromyography (sEMG) is an important tool in many therapeutic and rehabilitation applications of the shoulder. Surface EMG provides non-invasive information on the amplitude and timing of muscle activity as well as muscle fatigue (De Luca 1997). Many muscle- and subject-specific factors affect the sEMG signal, making it essential to normalize the signal when comparing between individuals or muscles (Veiersted 1991; Mathiassen et al. 1995; De Luca 1997). Muscle activity is most often normalized to maximal activation found using isometric maximum voluntary exertions (MVE) in specific postures and direction of exertion for each muscle.

The muscles of the shoulder complex are challenging to obtain MVEs for normalizing because of their number and multiple functions. Multiple MVEs for each of the shoulder muscles can cause pain and discomfort, tissue trauma, delayed soreness, and are very time-consuming during experimental protocols (Veiersted 1991; Bao et al. 1995; Mathiassen et al. 1995). Repeated maximal exertions can also lead to muscle fatigue, which can be identified through increases in sEMG amplitude and decreases in frequency. To reduce the number of MVEs required, tests to elicit maximum activation from multiple muscles simultaneously have been used. Kelly et al. (1996), concluded that 4 of the 27 exertions they tested were necessary to maximally activate the 8 shoulder muscles examined; however, they did not compare these results to individual muscle (IM) tests. Maximum activation is more dependent on exertion direction than posture itself (Chopp et al. 2010), making this an important limitation in the application of this work. Boettcher et al. (2008), also developed a protocol of four tests to elicit maximal activity from a large subset of the shoulder muscles. Although they examined many postures and exertion directions, they did not include traditional IM tests in their design. Previous work examined the utility of combining multiple and single muscle tests, and although they have found this method to be successful, only a subset of shoulder muscles were examined (Chopp et al. 2010; Rota et al. 2013). Attempts to expand this four test protocol to include rhomboid major and teres major demonstrated IM tests elicited greater activation levels for these muscles (Ekstrom et al. 2005). However, research to date has not provided insight into how activation levels differ between multi-muscle and IM tests.

The purpose of this investigation was to compare maximum activity elicited from a previously published multi-muscle test MVE protocol (Boettcher et al. 2008) against a protocol of IM MVE tests. Furthermore, we aimed to evaluate the reliability of these protocols, and to determine the MVE test protocol, consisting of both multi-muscle and IM tests, that should be used in future shoulder investigations. We hypothesized that the two test protocols (IM and multi-muscle) would elicit comparable maximum values and would be reliable.

## Methods

**Figure 1.**
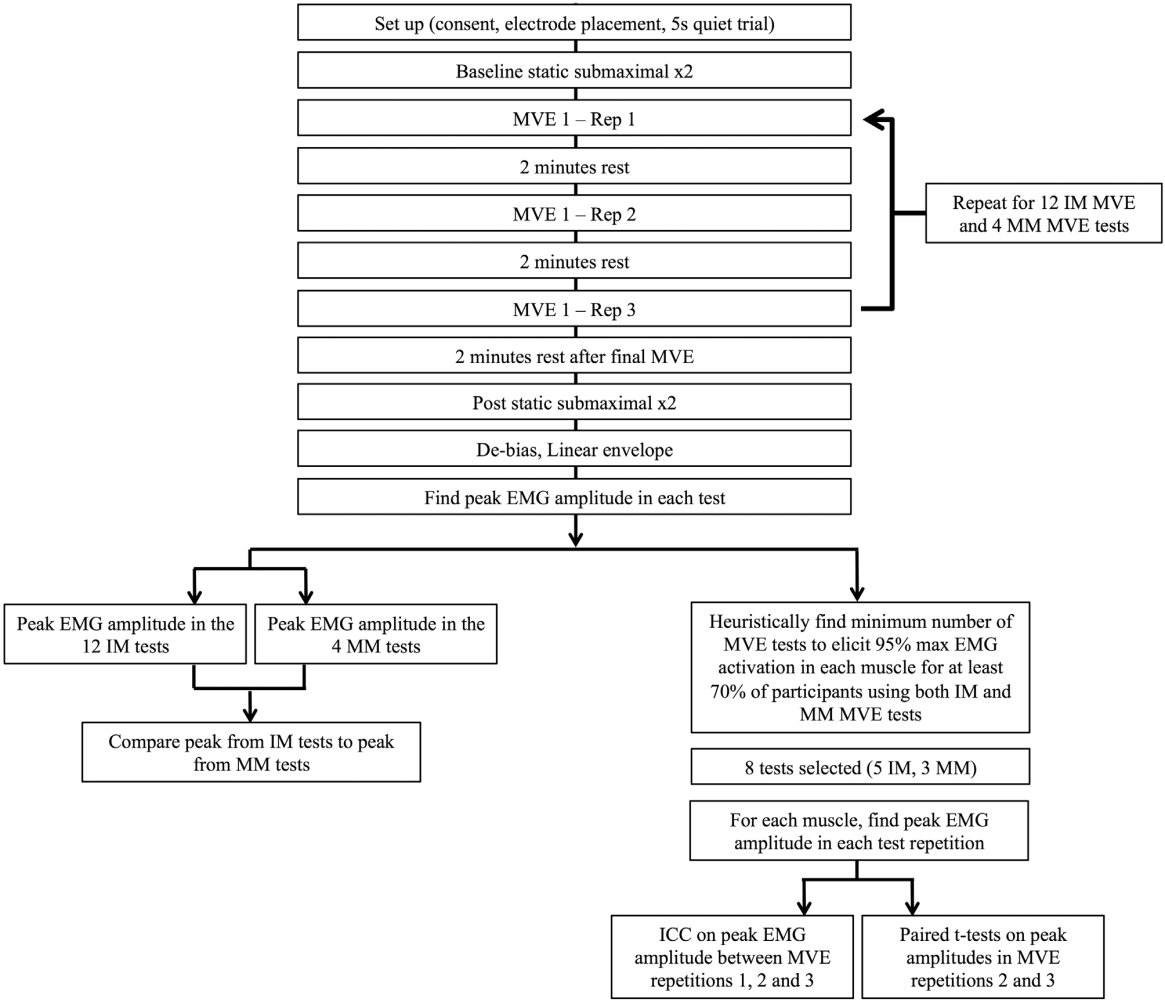
Overview of study protocol and analyses. IM = individual muscle test, MM = multi-muscle test.

Ten right-handed men participated in the laboratory study (23.6 ± 3.4 years, 179.0 ± 4.8 cm, 79.4 ± 12.6 kg), this sample size is consistent with previous literature (Yang & Winter 1983). All participants were free from upper extremity pathologies within the last year and recruited from the university population. The study was approved by the Hamilton Integrated Research Ethics Board. Participants provided informed, written consent prior to participation, completed the protocol in a single visit and were free to withdraw from the study at any time. The protocol and analyses are outlined in Figure 1.

Muscle activity was recorded from 12 right trunk, and upper extremity muscles (anterior, middle and posterior deltoids, infraspinatus, supraspinatus, upper, middle and lower trapezius, latissimus dorsi, serratus anterior and the clavicular and sternal heads of the pectoralis major) using a wireless surface EMG system (Trigno, Delsys Inc., Natick, MA, USA). Electrodes sites were located with guidance from the literature (Ekstrom et al. 2005; Waite et al. 2010; Hodder & Keir 2013) and confirmed with manual palpation. Sites were shaved and cleansed with isopropyl alcohol prior to electrode placement. Electrodes were oriented parallel to muscle fibres. EMG signals were sampled at 2000 Hz, differentially amplified (input impedance 10^15^Ω, CMRR > 80 dB), band-pass filtered (20–450 Hz), and converted with a 16-bit card with a ±5 V range.

Following electrode placement, a five-second quiet trial was collected. To evaluate muscle fatigue development in the anterior and middle deltoid muscles, participants performed two static submaximal reference exertions. Participants elevated their arm (1) to 90° in the sagittal plane, and (2) to 90° in the frontal plane, supporting only the weight of their arm. In these postures, a hand load is not required to elicit muscle activity over 15–20% MVE (Öberg et al. 1994; Antony & Keir 2010). Following the reference exertions, participants performed 12 IM, and 4 multi-muscle MVEs (Table 1). Each MVE was repeated 3 times for a total of 48 maximal exertions. Postures were confirmed with a manual goniometer. Each 5 s MVE was followed by 2 min of rest between repetitions of the same MVE. No rest was given between MVEs for different muscles (e.g. moving between the MVE for anterior deltoid and posterior deltoid), and order of exertions was block randomized for each participant. Two minutes following the final maximum exertion, the two submaximal reference exertions were repeated to evaluate muscle fatigue over the course of the protocol. Participants did not report any pain during the MVE protocol.

**Table 1.** Test postures and exertions for the individual muscle (IM) and the multi-muscle (MM) test protocols (Ekstrom et al. 2005; Dark et al. 2007; Boettcher et al. 2008; Waite et al. 2010; Hodder & Keir 2013). Test postures were confirmed with a manual goniometer.

Test	Posture	Exertion
Anterior deltoid (IM)	Straight arm with 45° flexion	Shoulder flexion with resistance at wrist
Middle deltoid (IM)	Straight arm with 45° abduction	Shoulder abduction with resistance at wrist
Posterior deltoid (IM)	Shoulder 90° abduction; Elbow 90°	Horizontal extension with resistance at elbow
Infraspinatus (IM)	Arm at side with 90° elbow flexion	External rotation with resistance at wrist
Supraspinatus (IM)	Straight arm with slight abduction	Abduct
Upper trapezius (IM)	Arm abducted to 90° with neck side-bent, rotated to the opposite side and extended	Abduct
Middle trapezius (IM)	Abduct 120°, thumb pointing backward	Exert backwards/lateral scapular rotation
Lower trapezius (IM)	Arm abducted to 90°, elbow flexed to 90°	Squeeze scapula together with resistance proximal to the humerus
Latissimus dorsi (IM)	Arm abducted 90°, elbow flexed to 90°	Shoulder adduction and extension with resistance under elbow
Serratus (IM)	Arm abducted 90°, elbow 180°	Push forward in horizontal flexion
Pectoralis major sternal head (IM)	Shoulder ~90°; Elbow ~90°	Bilateral palm press
Pectoralis major clavicular head (IM)	Shoulder 90° flexion; Elbow 90°	Horizontal (axial plane) adduction with resistance proximal to elbow
Empty can (MM)	Arm abducted 90°, 30° cross flexion, humerus internally rotated (thumb pointing down)	Flex and abduct with resistance at wrist
125° Flexion (MM)	Arm flexed 125°, forearm pronated, elbow 180°	Flexion with resistance above elbow, pressure on inferior angle of scapula
Palm press (MM)	Arm flexed 90°, elbow 20°, forearm semi prone	Horizontal adduction, resistance at heel of palm
Internal rotation (MM)	Arm abducted 90°, cross flex 30°, elbow 90°	Internal rotation with residence at wrist

### Data analysis

Bias was removed from raw sEMG data by subtracting the mean of the quiet trial for each muscle. The sEMG data were linear enveloped with a 2nd order, 4 Hz dual-pass Butterworth filter. Single peak values for each muscle were extracted from each test and used in subsequent evaluations. To quantify fatigue development, the median power frequency (MDF) of the middle and anterior deltoid muscles was calculated from a middle 3-s window in each of the pre- and post-test submaximal trials using the raw sEMG signal. Myoelectric fatigue was defined as an 8% decrease in the MDF from the pre- to the post-test exertion (Öberg et al. 1990).

The sEMG data were initially divided into two sets for the analysis, peak amplitudes for each muscle were calculated from the 4 multi-muscle tests and from the 12 IM tests. Paired sample t-tests were used to compare each muscle’s maximum sEMG amplitude from the multi-muscle tests with the maximum amplitude from the IM tests. Within each test set (IM test set and multi-muscle test set), the maximum amplitude obtained for each muscle in any of the three repetitions was used, regardless of which specific MVE test or repetition elicited the value.

The specific MVE test that elicited the peak amplitude for each muscle varied between individual participants, thus based on preliminary results, a *post hoc* criterion was developed heuristically to select a minimum number of tests applicable to the most participants. This criterion was based on a trade off between minimizing the number of tests required and maximizing muscle activity across all of the included muscles. The criteria ensured the set of tests selected obtained 95% of each muscle’s maximum amplitude for at least 70% of participants. Once the optimal set of tests were selected, reliability was evaluated using Interclass Correlation Coefficients (ICC, two-way random effects model) and the set of tests selected were analyzed collectively for their reliability. Reliability was assessed between repetitions two and three only, allowing participants to use the first repetition of the test to familiarize themselves with the required action. Paired t-tests were used to evaluate differences between the maximum activities obtained in the second and the third repetitions of each test. All statistical tests were conducted in SPSS (v20.0, IBM, NY, USA) with *α* = 0.05.

## Results

IM specific tests elicited 29–60% greater activation for the infraspinatus, posterior deltoid and latissimus dorsi muscles (*p* < 0.05) than the set of multi-muscle (MM) MVE tests. For the remaining nine muscles, there were no statistically significant differences in the maximum sEMG amplitudes obtained from the two sets of tests (IM and MM) (*p* > 0.05), however, these maximum sEMG amplitudes differed by 6–15% (Table 2).

**Table 2.** Maximum EMG voltage for each muscle from the 12 individual muscle tests (IM) and the 4 multi-muscle tests (MM). Muscles with significantly different mean values are denoted with *. The muscles % difference values that are less than 100% had greater max values with the individual muscle (IM) tests and those that are greater than 100% had greater values from the multi-muscle (MM) tests.

Muscle	Test	Mean (V)	SD	MM max as a % of IM max (%)
Anterior deltoid (AD)	IM	0.215	0.095	87
	MM	0.188	0.075	
Middle deltoid (MD)	IM	0.129	0.087	91
	MM	0.117	0.053	
Posterior deltoid* (PD)	IM	0.217	0.091	71
	MM	0.154	0.064	
Infraspinatus* (IN)	IM	0.112	0.077	70
	MM	0.079	0.059	
Supraspinatus (SU)	IM	0.169	0.113	91
	MM	0.154	0.105	
Upper trapezius (UT)	IM	0.117	0.073	85
	MM	0.100	0.067	
Middle trapezius (MT)	IM	0.123	0.097	89
	MM	0.110	0.085	
Lower trapezius (LT)	IM	0.100	0.074	89
	MM	0.090	0.072	
Latissimus doris* (LD)	IM	0.047	0.019	40
	MM	0.019	0.011	
Serratus anterior (SA)	IM	0.158	0.152	106
	MM	0.168	0.155	
Pectoralis major sternal (PS)	IM	0.055	0.038	108
	MM	0.060	0.037	
Pectoralis major clav (PC)	IM	0.081	0.045	111
	MM	0.090	0.065	

**Figure 2.**
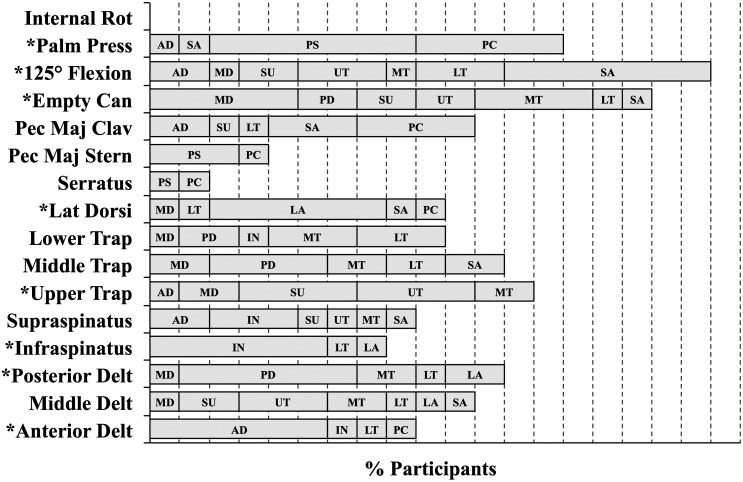
Percent of participants that achieved at least 95% of the listed muscles maximum amplitude with each test. Each vertical dashed line represents 10% of the participants. Tests marked with * are the ones selected for the recommended test protocol for future work. For example, using the palm press test, anterior deltoid was activated to greater than or equal to 95% of maximum in 10% of participants, serratus anterior in 10%, pectoralis major sternal 70% and pectoralis major clavicular in 60% of participants.

**Figure 3.**
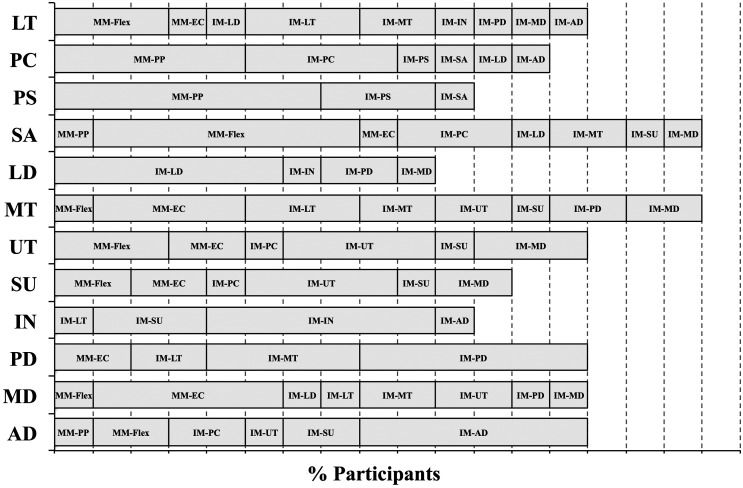
The percent of participants that had at least 95% of the listed muscles maximum amplitude with the listed tests. Each vertical dashed line represents 10% of the participants. For example, the latissimus dorsi was activated to greater than or equal to 95% of maximum activation for 60% of participants in the IM-LD test, 10% in the IM-IN test, 20% in the IM-PD test and 10% of participants in the IN-MD test.

**Figure 4.**
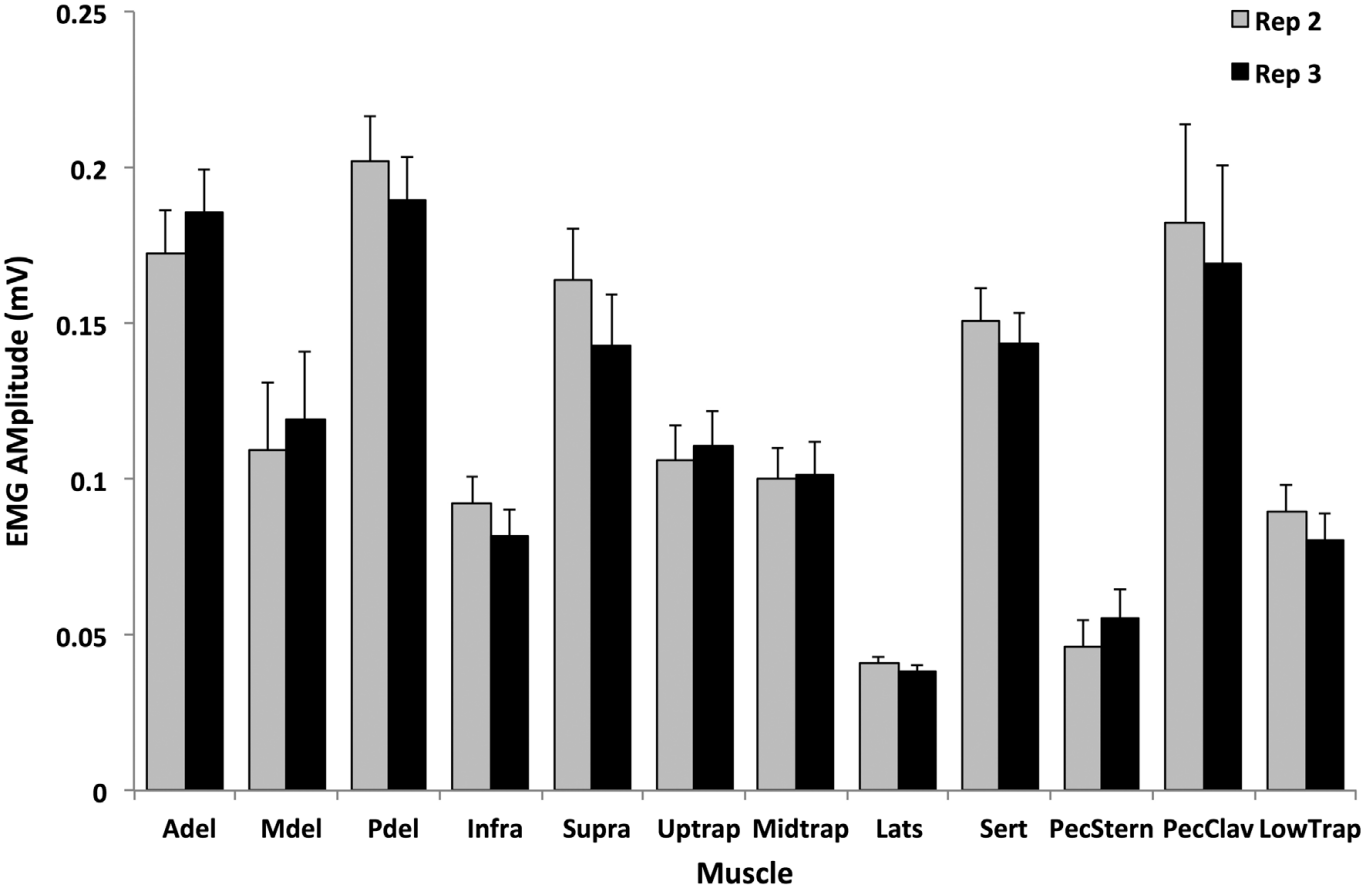
Comparison of maximum sEMG amplitude between the second and third repetitions of the recommended eight test protocol for each muscle. Reliability of maximum sEMG amplitude (V) between the second and third repetitions. There were no statistically significant differences in the maximum values elicited between the second (grey bars) and third (black bars) repetitions of the protocol (*p* > 0.05). Error bars depict the within subject standard deviations between the two repetitions.

A set of eight tests was selected from the 16 exertions that obtained 95% of each muscle’s maximum for at least 70% of the participants (Figure 2). These tests included the individual tests for the anterior and posterior deltoids, infraspinatus, latissimus dorsi and upper trapezius muscles and the 125° flexion, empty can and palm press multi-muscle tests (Table 1). The specific test that elicited the maximum amplitude for each muscle varied between participants (Figure 3). ICC between the three repetitions of each muscle’s maximum amplitude in the set of eight tests listed above ranged from 0.831 to 0.986 (excellent according to Fleiss 1986) (Table 3). Paired *t*-tests showed that there were no significant differences between the maximum amplitudes obtained in the second and third repetitions of the tests (*p* > 0.05) (Figure 4).

**Table 3.** ICC values (mean measures) and 95% confidence intervals showing the reliability between the three repetitions of each muscle’s maximum amplitude in the set of eight tests.

Muscle	ICC (Mean measures)	95% confidence interval	
		Lower bound	Upper bound
Anterior deltoid	0.966	0.902	0.991
Middle deltoid	0.952	0.858	0.987
Posterior deltoid	0.979	0.939	0.994
Infraspinatus	0.983	0.95	0.995
Supraspinatus	0.986	0.959	0.996
Upper trapezius	0.97	0.912	0.992
Middle trapezius	0.985	0.955	0.996
Lower trapezius	0.993	0.979	0.998
Latissimus dorsi	0.979	0.939	0.994
Serratus anterior	0.995	0.985	0.999
Pectoralis major-clavicular	0.995	0.986	0.999
Pectoralis major-sternal	0.936	0.814	0.983

No signs of myoelectric fatigue were found in the submaximal reference contractions of anterior or middle deltoid muscles following the 48 maximum exertions (less than 8% decrease in MDF (Öberg et al. 1990)).

## Discussion

The aims of this investigation were to determine if a previously published set of four multi-muscle MVE tests could effectively replace individual shoulder muscle MVE tests for a more time efficient and safe protocol for experimental studies, and to examine the reliability of these values between multiple repetitions of each exertion. The statistical analysis revealed few differences between the two test sets (IM and MM), however, large differences in the actual values suggest that this four MVE test protocol might have limitations in practical and research applications. To develop a protocol that would elicit maximum activation in all muscles in the fewest number of tests, a heuristic *post hoc* analysis was completed to select a set of individual and multi-muscle MVE tests. Reliability between multiple repetitions of the exertions was found to be excellent and that participants were able to complete 48 maximum exertions within 1 test session without developing signs of myoelectric fatigue when given 2 min of rest between each exertion.

Although there were few statistical differences between the two test sets (12 IM vs. 4 MM tests), we found differences of 6–15% MVE between statistically non-significant values. Underestimating maximum activity by as little as these statistically non-significant levels can lead to overestimating submaximal sEMG amplitude, which, depending on the amplitude and the research question or ergonomic assessment goals, may lead to a functional difference or increased variability inherent to the normalization process (Yang & Winter 1983). For example, Jonsson (1978), recommended that static submaximal work not exceed 2–5% MVC, thus even small overestimations of muscle activations can have significant implications in the design and evaluation of return to work task assessments. Using upper extremity muscles, including a subset of the shoulder muscles, previous work found a combination of strength exercises and IM tests was the best way to normalize sEMG data (Rota et al. 2013). Investigations involving the shoulder complex often require a larger number of shoulder muscles than included in Rota et al. (2013); the current investigation expands this literature by recommending a set of 8 tests to normalize 12 shoulder muscles.

Selecting only one test for each muscle may be challenging due to between subject variability in shoulder muscle activation patterns (Nieminen et al. 1993), which was confirmed in our study as demonstrated by the between subject differences in which MVE tests elicited the maximum amplitude for each muscle (Figure 3). The multi-muscle tests elicited high sEMG amplitudes; however, because of between subject variability and large differences in each IMs function, these tests alone were not sufficient to elicit maximum sEMG amplitude from all muscles examined. Although 3 of the 4 multi-muscle tests evaluated (empty can, palm press, 125° flexion) were selected as part of the 8 test set, reducing the protocol to only 4 tests may prevent finding true maximum activity level, while 12 individual tests may prove too lengthy. The proposed 8 MVE tests (empty can, palm press, 125° flexion, anterior deltoid, posterior deltoid, latissimus dorsi, upper trapezius, infraspinatus) represent a trade-off between the convenience of 4 multi-muscle tests and the specificity of 12 IM tests. This proposed test protocol produced higher maximum amplitudes than either of the other two test sets alone. Although the criterion used to select the 8-test set did not require maximal activation for every muscle in all participants, it performed better than the IM tests and multi-muscle tests alone. Depending on the muscle, the proportion of participants that obtained their maximum with this 8 test set ranged from 50 to 90%. Alternatively, 30–100% of the participants reached their maximum in the IM protocol while 0–70% of participants reached their maximum with the multi-muscle protocol. The combination of multi-muscle and IM tests elicited greater muscle activation from all the test muscles, compared to either the multi-muscle or the IM tests alone.

The reliability of the maximum amplitude from of the eight recommended tests was excellent, suggesting two repetitions of each test is sufficient for obtaining maximum sEMG amplitude. Previous work has shown that shoulder MVE repeatability was dependent on the muscle tested (Fischer et al. 2011). The ICCs found in this investigation ranged from 0.831 to 0.986. According to Fleiss (1986), these would be considered excellent (0–0.4 weak, 0.4–0.75 fair to good and greater than 0.75 excellent). This shows that the recommended protocol elicited reliable maximum amplitude from all of the muscles included in the investigation with only two repetitions of each test. Collecting MVEs for every muscle is very time consuming in shoulder research because of the large number of muscles typically included in these investigations. By reducing the number of repetitions for each test from 3 to 2, we are able to reduce the time of the MVE portion of the data collections for 2–3 min/muscle tested, which can have a sizable impact in shoulder muscle assessment, rehabilitation and research. It was confirmed with EMG amplitude and frequency, that the 48 MVE protocol did not elicit muscle fatigue when 2 min rest was given between each exertion. Although this protocol focused on shoulder muscles, the recommendation of 2 min of rest between exertions can be applied to different muscle groups.

There are limitations to the current investigation. The order of the MVE tests were randomized for each participant and it is possible that two tests targeting the same muscle could have directly followed each other. We controlled order effects by randomizing the test order between participants and confirmed that fatigue did not develop with this protocol by evaluating changes in MDF during submaximal exertions before and after the MVE protocol. Although there were several different options for shoulder muscle MVE tests in the published literature, a set of 16 tests was selected for this investigation. Since participants were completing 48 maximum exertions with the current protocol, we were not able to include and evaluate multiple tests for each muscle. Only healthy males were included in this investigation and future investigations are required to confirm that this test protocol is appropriate for female participants and clinical populations as well.

## Conclusion

Two repetitions each of eight tests (empty can, palm press, 125° flexion, anterior deltoid, posterior deltoid, latissimus dorsi, upper trapezius, infraspinatus) are recommended to effectively generate repeatable, maximal muscle activation in the examined 12 shoulder muscles using sEMG. The findings also show that, with two minutes of rest between each maximum exertion participants are able to complete at least 48 maximum exertions without signs of myoelectric fatigue. This shoulder muscle normalizing protocol can be incorporated into other experimental protocols to elicit maximum sEMG amplitude in a more time efficient manner than previous protocols.

## Disclosure statement

No potential conflict of interest was reported by the authors.

## Funding

This work was supported by the Natural Sciences and Engineering Research Council of Canada [grant number DG 217382-09, PGS-D] and Mitacs Elevate PDF.

## References

[CIT0001] Antony NT, Keir PJ 2010 Effects of posture, movement and hand load on shoulder muscle activity. J Electromyogr Kines. 20:191–198. 10.1016/j.jelekin.2009.04.010 19473855

[CIT0002] Bao S, Mathiassen SE, Winkel J 1995 Normalizing upper trapezius EMG amplitude: comparison of different procedures. J Electromyogr Kines. 5:251–257. 10.1016/1050-6411(95)00011-9 20719656

[CIT0003] Boettcher CE, Ginn KA, Cathers I 2008 Standard maximum isometric voluntary contraction tests for normalizing shoulder muscle EMG. J Orthop Res. 26:1591–1597. 18528827 10.1002/jor.20675

[CIT0004] Chopp JN, Fischer SL, Dickerson CR 2010 On the feasibility of obtaining multiple muscular maximum voluntary excitation levels from test exertions: a shoulder example. J Electromyogr Kines. 20:896–902. 10.1016/j.jelekin.2009.10.002 19879776

[CIT0005] Dark A, Ginn KA, Halaki M 2007 Shoulder muscle recruitment patterns during commonly used rotator cuff exercises: an electromyography study. Phys Ther. 87:1039–1046. 10.2522/ptj.20060068 17578940

[CIT0006] De Luca CJ 1997 The use of surface electromyography in biomechanics. J Appl Biomech. 13:135–163. 10.1123/jab.13.2.135

[CIT0007] Ekstrom RA, Soderberg GL, Donatelli RA 2005 Normalization procedures using maximum voluntary isometric contractions for the serratus anterior and trapezius muscles during surface EMG analysis. J Electromyogr Kines. 15:418–428. 10.1016/j.jelekin.2004.09.006 15811612

[CIT0008] Fischer SL, Grewal TJ, Wells R, Dickerson CR 2011 Effect of bilateral versus unilateral exertion tests on maximum voluntary activity and within-participant reproducibility in the shoulder. J Electromyogr Kines. 21:311–317. 10.1016/j.jelekin.2010.05.002 20542446

[CIT0009] Fleiss JL 1986 The design and analysis of clinical experiments. Toronto: Wiley.

[CIT0010] Hodder JN, Keir PJ 2013 Obtaining maximum muscle excitation for normalizing shoulder electromyography in dynamic contractions. J Electromyogr Kines. 23:1166–1173. 10.1016/j.jelekin.2013.06.012 23871651

[CIT0011] Jonsson B 1978 Kinesiology-with special reference to electromyographic kinesiology. Contemp Clin Neurophysiol EGG Suppl No. 34:417–428. 285846

[CIT0012] Kelly BT, Kadrmas WR, Kirkendall DT, Speer KP 1996 Optimal normalization tests for shoulder muscle activation: An electromyographic study. J Orthop Res. 14:647–653. 10.1002/(ISSN)1554-527X 8764876

[CIT0013] Mathiassen SE, Winkel J, Hägg GM 1995 Normalization of surface EMG amplitude from the upper trapezius muscle in ergonomic studies-A review. J Electromyogr Kines. 5:197–226. 10.1016/1050-6411(94)00014-X 20719652

[CIT0014] Nieminen H, Takala EP, Viikari-Juntura E 1993 Normalization of electromyogram in the neck-shoulder region. Eur J Appl Physiolo. 67:199–207. 10.1007/BF00864215 8223530

[CIT0015] Öberg T, Sandsjö L, Kadefors R 1990 Electromyogram mean power frequency in non-fatigued trapezius muscle. Eur J Appl Physiolo. 61:362–369. 10.1007/BF00236054 2079054

[CIT0016] Öberg T, Sandsjö L, Kadefors R 1994 Subjective and objective evaluation of shoulder muscle fatigue. Ergonomics. 37:1323–1333. 7925256 10.1080/00140139408964911

[CIT0017] Rota S, Rogowski I, Champely S, Hautier C 2013 Reliability of EMG normalization methods for upper-limb muscles. J Sport Sci. 31:1696–1704. 10.1080/02640414.2013.796063 23697512

[CIT0018] Veiersted KB 1991 The reproducibility of test contraction for calibration of electromyographic measurements. Eur J Appl Physiolo. 62:91–98. 10.1007/BF00626762 2022209

[CIT0019] Waite DL, Brookham RL, Dickerson CR 2010 On the suitability of using surface electrode placements to estimate muscle activity of the rotator cuff as recorded by intramuscular electrodes. J Electromyogr Kines. 20:903–911. 10.1016/j.jelekin.2009.10.003 19932033

[CIT0020] Yang JF, Winter DA 1983 Electromyography reliability in maximum and submaximum isometric contractions. Arch Phys Med Rehabil. 64:417–420. 6615179

